# Supplementary figures and images for: Multiple knockout mutants reveal a high redundancy of phytotoxic compounds contributing to necrotrophic pathogenesis of Botrytis cinerea
Source: PLoS Pathog. 2022 Mar 3;18(3):e1010367. doi: 10.1371/journal.ppat.1010367 (PMC8923502; doi:10.1371/journal.ppat.1010367)

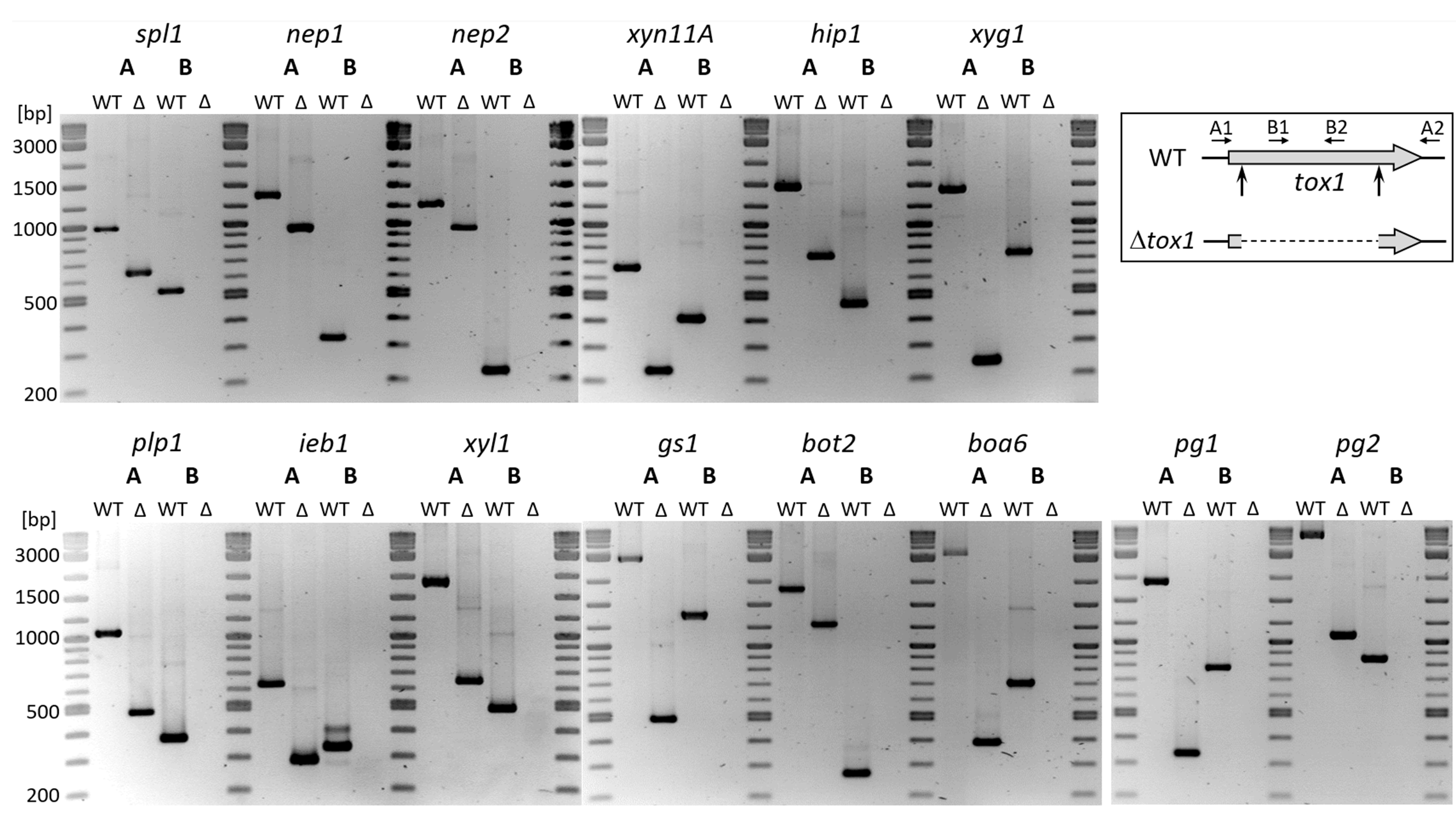

Supplement: S2 Fig — Position of primer pairs used are indicated in the sketch. A: PCR with primers A1/A2 flanking the deleted region, to verify the the CRISPR/Cas9-NHEJ-induced deletions. B: PCR with primers B1/B2 amplifying an internal part of the deleted region, to confirm the absence of any WT DNA in the purified mutants. Missing PCR products in reactions B confirm homokaryosis of deletion mutants. The sizes of the deletions were determined by Sanger and genome sequencing (S2 Table). Primers used are shown in S1 Table. (TIF) [file ppat.1010367.s002.tif]

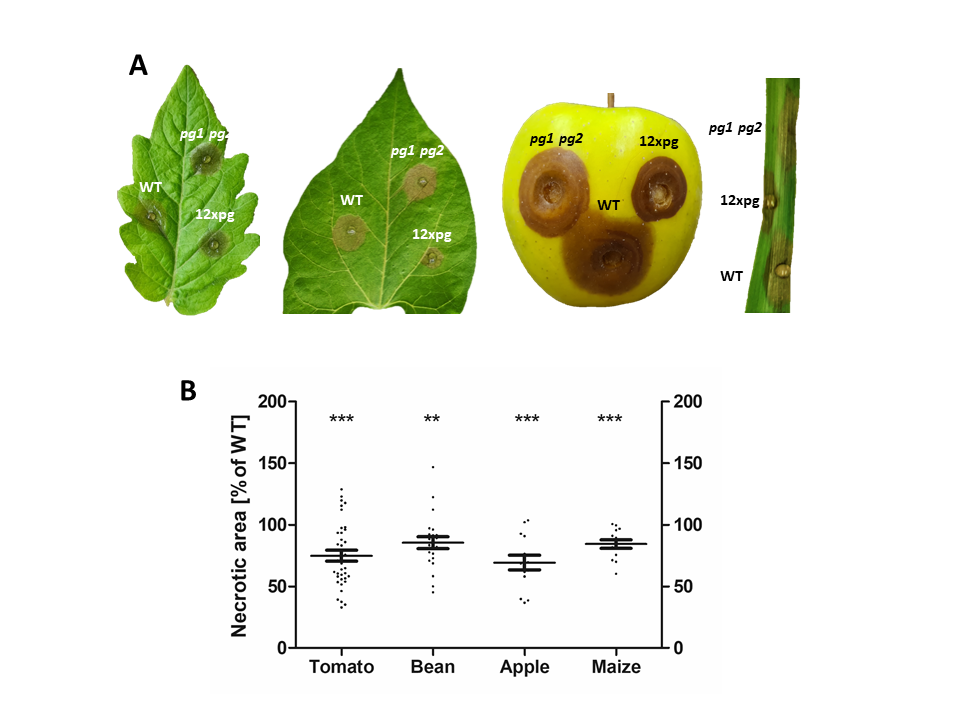

Supplement: S3 Fig — A: Tomato leaf (48 h.p.i.), bean leaf (attached, 48 h.p.i.), apple fruit (96 h.p.i.) and maize leaf (72 h.p.i.), infected with WT, pg1 pg2 and 12xpg mutants. B: Lesion formation of pg1 pg2 mutant (relative to WT) on different plant tissues. The p values by one-sample t test to a hypothetical value of 100% (WT) are indicated. **p < 0.01; ***p < 0.001. (TIF) [file ppat.1010367.s003.tif]

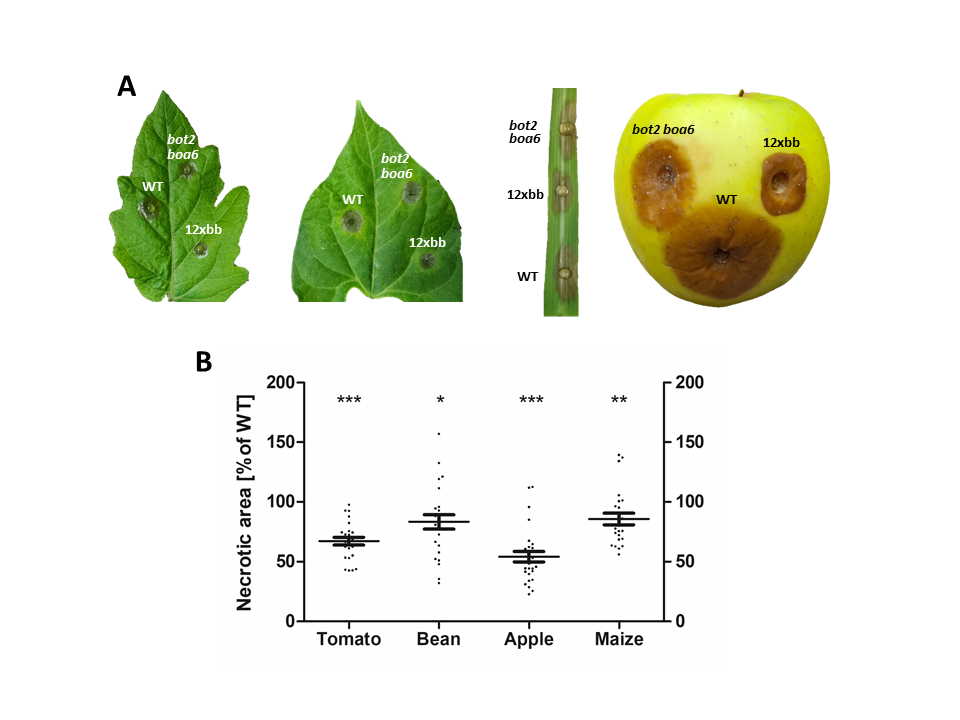

Supplement: S4 Fig — A: Tomato leaf (48 h.p.i.), bean leaf (attached, 48 h.p.i.), apple fruit (96 h.p.i.) and maize leaf (72 h.p.i.), infected with WT, bot2 boa6 and 12xbb mutants. B: Lesion formation of bot2 boa6 mutant (relative to WT). The p values by one-sample t test to a hypothetical value of 100% (WT) are indicated. *p < 0.05; **p < 0.01; ***p < 0.001. (TIF) [file ppat.1010367.s004.tif]

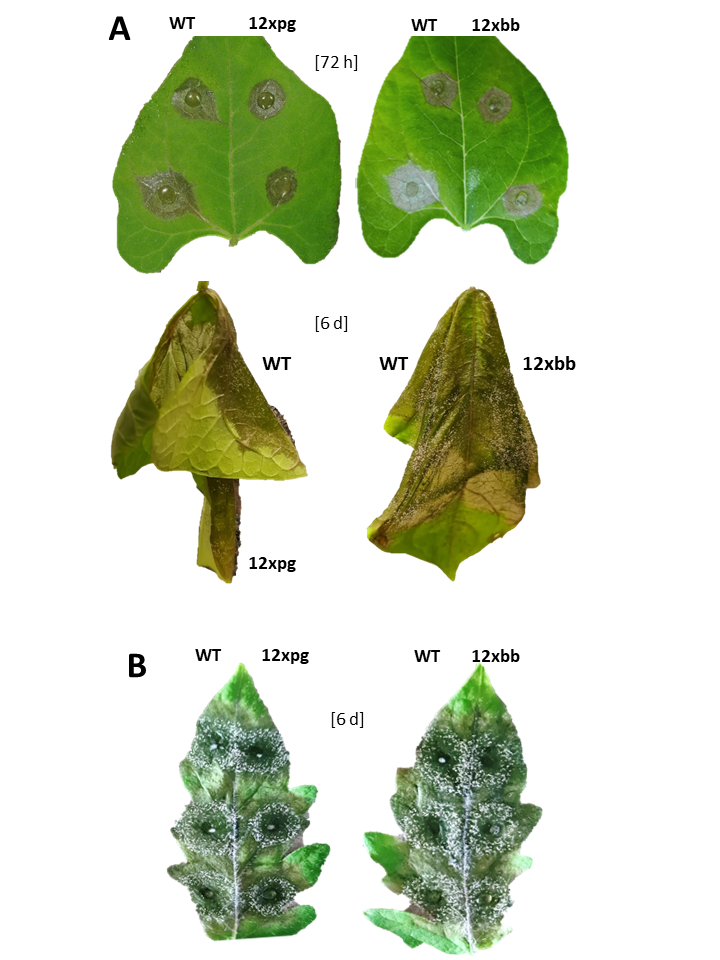

Supplement: S5 Fig — Lesion formation and sporulation of B. cinerea WT, 12xpg and 12xbb mutants on attached Phaseolus bean leaves (A), and on detached tomato leaves (B). (TIF) [file ppat.1010367.s005.tif]
