# Supplementary material for: Multiple knockout mutants reveal a high redundancy of phytotoxic compounds contributing to necrotrophic pathogenesis of Botrytis cinerea
Source: PLoS Pathog. 2022 Mar 3;18(3):e1010367. doi: 10.1371/journal.ppat.1010367 (PMC8923502; doi:10.1371/journal.ppat.1010367)
Supplement: S1 Table — (DOCX) [file ppat.1010367.s006.docx]

**S1 Table. Oligonucleotides used**

| **Primer Name** | **Sequence** | **Use** |
| --- | --- | --- |
| Gib_pTEL_S_EcorV_PtrpC | AGCTGATGATCACTTAAGAACGCGTAGATCATTTTTTGGGCTTGGCTGG | Amplification of P*trpC* |
| PtrpC_ol_CypR_RV | CCCTCGGAAACATTTGGATGCTTGGGTAGAATAGGT |  |
| CypR_ol_Ptrp_FW | CCCAAGCATCCAAATGTTTCCGAGGGTGTTGCC | Amplification of CypR |
| CypR_ol_TniaD_RV | CTCTCAGTTACTTATTCTTCGCCAAAAGGATGGTTGAACTTCAGGAACC |  |
| TniaD_ol_CypR_FW | ACCATCCTTTTGGCGAAGAATAAGTAACTGAGAGGTGGTTTAGATATGG | Amplification of T*niaD* |
| Gib_pTEL_S_EcorV_TniaD | CAATTCCTAGGTCTAGATGCATGCAGATCTCACTGATACATCTGGCACC |  |
| xyn11A_KO-Fl_l_FW | CCCCCTCGAGGTCGACGGTATCGATAAGCTTGATCCCTCACATCAAGTGTATGTGAT | Amplification of left flank for *xyn11A* knockout |
| xyn11A_KO-Fl_l_RW | CATGCATGTGAGCAAAAGGCCAGCAAAGTTGATTGTAGTAGAGTATGTAGATGAGAT |  |
| xyn11A_KO-Fl_r_FW | CATTTGCGGGTCCTTTCCGGCGATCGACGGTTCCACATACAAGATCC | Amplification of right flank for *xyn11A* knockout |
| xyn11A_KO-Fl_r_RW | AGAACTAGTGGATCCCCCGGGCTGCAGGAATTCGATAGAAATGGGTGATGAGTATGTAGGTTATT |  |
| R-Casette_FW | TGCTGGCCTTTTGCTCACATGCATG | Amplification of resistance cassettes from pTEL vectors |
| R-Casette_RV | ATCGCCGGAAAGGACCCGCAAATG |  |
| xyg1_KO_left_FW | CCCCCTCGAGGTCGACGGTATCGATAAGCTTGATGAATTGCCAAAGTACAGCTTCAAACTTCTA | Amplification of left flank for *xyg1* knockout |
| xyg1_KO-left_RW | CATGCATGTGAGCAAAAGGCCAGCAGAATGTTTGAATGAAGGAAAAGAAGAGCG |  |
| xyg1_KO-right_FW | CATTTGCGGGTCCTTTCCGGCGATGATGCGAATCTATCTAAAGAGTGATATTGT | Amplification of right flank for *xyg1* knockout |
| xyg1_KO-right_RW | AGAACTAGTGGATCCCCCGGGCTGCAGGAATTCGATAATTTACGCGAGGGGAAGCTT |  |
| ieb1_KO-left_FW | CCCCCTCGAGGTCGACGGTATCGATAAGCTTGATCCTTGTACCCTTTGTGCATAGCTAG | Amplification of left flank for *ieb1* knockout |
| ieb1_KO-left_RW | CATGCATGTGAGCAAAAGGCCAGCATTGTGTATTTGGTTGCTTGATATGAAGAT |  |
| ieb1_KO-right_FW | CATTTGCGGGTCCTTTCCGGCGATTGATTAGTTCCTTGGCGACATTGC | Amplification of right flank for *ieb1* knockout |
| ieb1_KO-right_RW | AGAACTAGTGGATCCCCCGGGCTGCAGGAATTCGATGGTGACATGAGCCTTGATTCTATGTATATTC |  |
| spl1_KO-left_FW | CCCCCTCGAGGTCGACGGTATCGATAAGCTTGATAATTCTCGTGTTGTTGTCGAACAAAC | Amplification of left flank for *spl1* knockout |
| spl1_KO-left_RW | CATGCATGTGAGCAAAAGGCCAGCAACATCATGTCTTATATACCTTCTACCCCTC |  |
| spl1_KO-right_FW | CATTTGCGGGTCCTTTCCGGCGATATAGGAAGTGGAAGAATGGGATAGG | Amplification of right flank for *spl1* knockout |
| spl1_KO-right_RW | AGAACTAGTGGATCCCCCGGGCTGCAGGAATTCGATGCTGAGGTGTAAGGAAATGGTGGA |  |
| nep2_KO-left_FW | CCCCCTCGAGGTCGACGGTATCGATAAGCTTGATCCGAATCACGAAAGAAAGTTTTGTAG | Amplification of left flank for *nep2* knockout |
| nep2_KO-left_RW | CATGCATGTGAGCAAAAGGCCAGCAACTACATTCTACAAGAGGAAACGCA |  |
| nep2_KO-right_FW | CATTTGCGGGTCCTTTCCGGCGATGGCTATATTCTGGGAGTCTATTGGC | Amplification of right flank for *nep2* knockout |
| nep2_KO-right_RW | AGAACTAGTGGATCCCCCGGGCTGCAGGAATTCGATATGTGAGAGGTTAATCGACATTTAGAATGAG |  |
| nep1 KO F | CAGCATCAACAGCATCAGCTTCCATTCCATATTCATTACATTCCACATTACCACTTTCGTATCGCCGGAAAGGACCCGCAAATG | Amplification of *nep1*-hygR  repair template |
| nep1 KO R | ACGATCTCTGACAGGACAAACTTCCAGATTCTCCAGAACTCTATCTAGTAAGAAAGAACTTGCTGGCCTTTTGCTCACATGCATG |  |
| nep2 KO F | CACACATCTATTAATCGCTCTCTCCTCTTAGTTACAAGGAAAATCCAGACAAACTTCATCTGCTGGCCTTTTGCTCACATGCATG | Amplification of nep2-fenR  repair template |
| nep2 KO R | GCAAAAGCCAATAGACTCCCAGAATATAGCCCCTTATATTCATACATAAAGACACAAAGTATCGCCGGAAAGGACCCGCAAATG |  |
| nep1 gRNA 1 | AAGCTAATACGACTCACTATAGCGCTTGGGTCAACAACCCCGGTTTTAGAGCTAGAAATAGCAAG | sgRNA synthesis for *nep1* |
| nep2 gRNA 1 | AAGCTAATACGACTCACTATAGTATGTTCGAGGAGGACAAAGGTTTTAGAGCTAGAAATAGCAAG | sgRNA synthesis for *nep2* |
| gRNA _reverse | AAAAGCACCGACTCGGTGCCACTTTTTCAAGTTGATAACGGACTAGCCTTATTTTAACTTGCTATTTCTAGCTCTAAAAC | Constant oligonucleotide for sgRNA synthesis |
| Uni KO fw | CATTTGCGGGTCCTTTCCGGCGAT | universal oligo for 3‘‑integration |
| Uni KO rev | CATGCATGTGAGCAAAAGGCCAGCA | universal oligo for 5‘‑integration |
| xyn11A_5´for | CCCTTTTGTGAAACAACCTGGA | Proof of integration  5‘-flank *xyn11A* |
| xyn11A_3´rev | GGAGATGTGACGGTGAGGATAT | Proof of integration  3‘-flank xyn11A |
| nep2_5´for | CATCTACCAGCCCACTCAGAT | Proof of integration  5‘-flank *nep2^N^* |
| nep2_3´rev | CTTCTCCTTTCATTCATGCATATCGC | Proof of integration  3‘-flank *nep2^N^* |
| pTrpC_rev | ACCGCCTGGACGACTAAAC | Proof of integration  5‘-flank *ieb1* |
| ieb1_5´for | GTCTCACAGTTGTCGCTGG | Proof of integration  5‘-flank *ieb1* |
| ieb1_3´rev | GACCGTCGAAAACGCAAAAG | Proof of integration  3‘-flank *ieb1* |
| spl1_5´for | ATTAACTTCGACGTCGTCGAC | Proof of Integration  5‘-flank *spl1* |
| spl1_3´rev | GTGGATGGAATTCTTGAACTCAGTC | Proof of integration  3‘-flank S*spl1* |
| xyg_5for | GTCAAGGTTGTACGACTTTATCCAT | Proof of Integration  5‘-flank *xyg1* |
| xyg_3rev | GTTCGAAGGGTTTGTCGATGA | Proof of integration  3‘-flank *xyg1* |
| WT_Screen_fw | GAGAATCACGACAATGCAAGA | Proof of Integration  5‘-flank *nep1^H^* |
| TL144 Nep1 seq R | CGTTGGCTTATTCAATGCGGAGG | Proof of integration  3‘-flank *nep1^H^* |
| TL145 Nep2 seq F | GAACTTTGAATAGTGGGCAGTTGGG | Proof of Integration  5‘-flank *nep2^F^* |
| TL146 Nep2 seq R | ACAAGGCGACCATGATTATTTCTGG | Proof of integration  3‘-flank *nep2^F^* |
| Bcxyn11A_KO_Confirm_F | TGAATGTCTTGCAAGAAAGAG | *xyn11A^N^* WT check |
| Bcxyn11A_KO_Confirm_R | ATTTAAGAAACAGTGATGGAAGC |  |
| Bcxyg_KO_Confirm_FW | GTTTCCTTAGTCTTGGCAACA | *xyg1^C^* WT check |
| Bcxyg_KO_Confirm_RW | GGTTGGCAGTCAAGTATGTAA |  |
| Bcspl1_q_F | CCACCCAAGGTTCCCTCAAG | *spl1^C^* WT check |
| BcsplI_KO_Confirm_RW | CTCCAACCATTTACAATCCACA |  |
| Bcnep2_KO-Confirm_FW | ATGGTTGCCTTCTCAAAATC | *nep2^N^* WT check |
| Bcnep2_KO-Confirm_RW | GGTTGTTTTCCACCAACAGTA |  |
| Bcieb1_KO_Confirm_FW | ATGTTCTCCAAGACCTTCATC | ieb1^C^ WT check |
| Bcieb1_KO_Confirm_RW | CCATTCTTGATTTAAGCGTACT |  |
| Bcnep1_KO-Confirm_FW | CATTTCTCCAACGCAAAATTC | *nep1^H^* WT check |
| Bcnep1_KO-Confirm_RW | GGAAGGAGACATAGTATTCGAC |  |
| Bcnep2_KO-Confirm_FW | ATGGTTGCCTTCTCAAAATC | *nep2^F^* WT check |
| Bcnep2_KO-Confirm_RW | GGTTGTTTTCCACCAACAGTA |  |
| pg1_ndeI_FW | CAACATATGACCGCAGCTCCAAACCC | *pg1* KO Check |
| pg1_EcoRI_RV | GTTGAATTCTTAACACTTGACACCAGATGGGAGACC |  |
| trpC-Screen | AGGAATCCGCTCTTGGCTCCAC | Proof of integration  3‘-flank *pg1* |
| natR-F | CTTCGTGGTCGTCTCGTACT | Proof of integration  3‘-flank *pg2* |
| PG_RTR | CCAGCACAAGCCTTCTTAAC |  |
| TL246 spl1-gRNA 1 | AAGCTAATACGACTCACTATAGATCATCGTAGCCAACGTCGTGTTTTAGAGCTAGAAATAGCAAG | Synthesis of *spl1* sgRNAs |
| TL247 spl1-gRNA 2 | AAGCTAATACGACTCACTATAGCAAGTCGACAAGAGTGCTTGGTTTTAGAGCTAGAAATAGCAAG |  |
| TL252_spl1_F | TGACAATGCACACAATGCGG | *spl1* KO check |
| TL253_spl1_R | TCCGAACATTCCGAGCTTCC |  |
| spl1_del-seq | CAAAATGCAATTCCCAACTC | *spl1* KO sequencing |
| spl1_wt_F | CCACCCAAGGTTCCCTCAAG | *spl1* WT check |
| spl1_wt_R | TCCGAACATTCCGAGCTTCC |  |
| TL238_Bcnep1_gRNA_3 | AAGCTAATACGACTCACTATAGATGGAGTCATGAGGAACGAGTTTTAGAGCTAGAAATAGCAAG | Synthesis of *nep1* sgRNAs |
| TL 167_Bcnep1_gRNA 2 | AAGCTAATACGACTCACTATAGACCTCCGACGACGTTTCCAGGTTTTAGAGCTAGAAATAGCAAG |  |
| TL143 Nep1 seq F | TCTGGTGCCGATTGAATACATCAAGTG | *nep1* KO check |
| TL144 Nep1 seq R | CGTTGGCTTATTCAATGCGGAGG |  |
| Bcnep1_KO-Confirm_FW | CATTTCTCCAACGCAAAATCC | *nep1* KO sequencing |
| TL267_Nep1_WT_F | CATGACTCCATCAACCCATGGG | *nep1* WT check |
| TL268_Nep1_WT_R | GAAAGGGATACTTACACCAGGCG |  |
| TL 169 Bcnep2 gRNA 2 | AAGCTAATACGACTCACTATAGTTACACCTTCCCAATCGTGAGTTTTAGAGCTAGAAATAGCAAG | Synthesis of *nep2* sgRNAs |
| TL241_Bcnep2_gRNA_4 | AAGCTAATACGACTCACTATAGTCGATGCATCGGGTAACACAGTTTTAGAGCTAGAAATAGCAAG |  |
| TL145 Nep2 seq F | GAACTTTGAATAGTGGGCAGTTGGG | *nep2* KO check |
| TL146 Nep2 seq R | ACAAGGCGACCATGATTATTTCTGG |  |
| Nep2_RT_fw | CAGTGGGACCGTAGGAACAG | *nep2* KO sequencing |
| TL269_Nep2_WT_F | CTCTTCCACACGATTGCTATTGAGTC | *nep2* WT check |
| Nep2_RT_rev | GGCTCGTCCTTTGGCATGTAC |  |
| TL243_BcXyn11a_gRNA_2 | AAGCTAATACGACTCACTATAGGTGGTGTTACATACTCCAAGTTTTAGAGCTAGAAATAGCAAG | Synthesis of *xyn11A* sgRNAs |
| TL245_BcXyn11a_gRNA_4 | AAGCTAATACGACTCACTATAGAAAATGGTTTGCAGTGGTGAGTTTTAGAGCTAGAAATAGCAAG |  |
| Bcxyn11A_KO_Confirm_FW | TGAATGTCTTGCAAGAAAGAG | *xyn11A* KO check |
| Bcxyn11A_KO_Confirm_RW | ATTTAAGAAACAGTGATGGAAGC |  |
| Bcxyn11A_KO-Confirm_FW | TGAATGTCTTGCAAGAAAGAG | *xyn11A* KO sequencing |
| TL285_WT_Xyn_F | GGTCAATATGCCGTAAGCTGGAC | *xyn11A* WT check |
| BcXylA.rv | CGTACGCTTGCTAGTACGGAC |  |
| TL288_Hip1_gRNA5 | AAGCTAATACGACTCACTATAGACAATGCCGATCTGTGGAGAGTTTTAGAGCTAGAAATAGCAAG | Synthesis of *hip1* sgRNAs |
| TL 163 HIP gRNA 3 GFP | AAGCTAATACGACTCACTATAGCTTGGACTACAGCTCCTAGGTTTTAGAGCTAGAAATAGCAAG |  |
| KO_Hip1_3'flank_seq | TGGCTAGTTTCTGGAGCTGTTG | *hip1* KO check |
| KO_Hip1_5'flank_seq | GTGATACATACTTGTCATGGGATG |  |
| KO_Hrp1_5'flank_seq | GTGATACATACTTGTCATGGGATG | *hip1* KO sequencing |
| Bcin14g01200_FW_NdeI | CAACATATGGCCATCGAGAAGCGCAGCAG | *hip1* WT check |
| Bcin14g01200_RV_EcoRI | TGTGAATTCCTAGGAGCTGTAGTCCAAGCCGAA |  |
| TL291_Xyg1_gRNA5 | AAGCTAATACGACTCACTATAGTTAAGGAACTGAGATGTGCGGTTTTAGAGCTAGAAATAGCAAG | Synthesis of *xyg1* sgRNAs |
| TL290_Xyg1_gRNA4 | AAGCTAATACGACTCACTATAGACTTTCTAAAATGCTCAACAGTTTTAGAGCTAGAAATAGCAAG |  |
| TL292_Xyg_F | TGAGCGCTCGTAACATGATATCCG | *xyg1* KO check |
| TL293_Xyg_R | TGAAATCTGGCCAACGTTCAAATCC |  |
| TL292_Xyg_F | TGAGCGCTCGTAACATGATATCCG | *xyg1* KO sequencing |
| Bcxyg_KO_Confirm_FW | GTTTCCTTAGTCTTGGCAACA | *xyg1* WT check |
| Bcxyg_KO_Confirm_RW | GGTTGGCAGTCAAGTATGTAA |  |
| TL310 sgRNA_RLP30_lig_1 | AAGCTAATACGACTCACTATAGATGGGAGAGATGATTTGAGGTTTTAGAGCTAGAAATAGCAAG | Synthesis of *plp1* sgRNAs |
| TL313 sgRNA_RLP30_lig_4 | AAGCTAATACGACTCACTATAGAGTGAGGTAGTGGTTGAAAGGTTTTAGAGCTAGAAATAGCAAG |  |
| TL306_RLP30_lig-F | ATCTTTCCATCGTCCCATAATCCC | *plp1* KO check |
| TL307_RLP30_lig-R | GGGTTTGGAGAACTCGTGATCG |  |
| TL307_RLP30_lig-R | GGGTTTGGAGAACTCGTGATCG | *plp1* KO sequencing |
| TL308_RLP30_lig_WT-F | CAGCACTCTTGCAACCGCTAC | *plp1* WT check |
| TL309_RLP30_lig_WT-R | GTTGTTCCAACTCCCGTGCTG |  |
| TL_259_Ieb1_gRNA4 | AAGCTAATACGACTCACTATAGAGCACGTGCAGAGACAATGGGTTTTAGAGCTAGAAATAGCAAG | Synthesis of *ieb1* sgRNAs |
| TL176_Bcieb1_gRNA_1 | AAGCTAATACGACTCACTATAGCACTCCGCCGATACCCACGGGTTTTAGAGCTAGAAATAGCAAG |  |
| Bcieb1_KO_Confirm_FW | ATGTTCTCCAAGACCTTCATC | *ieb1* KO check |
| Bcieb1_KO_Confirm_RW | CCATTCTTGATTTAAGCGTACT |  |
| Bcieb1_KO-Confirm_RW | CCATTCTTGATTTAAGCGTACT | *ieb1* KO sequencing |
| TL286_WT_Ieb_F | CTGCACGTGCTGCAAGC | *ieb1* WT check |
| TL287_WT_Ieb_R | GGGTATCGGCGGAGTGTGG |  |
| TL319_sgRNA_Xyl_2 | AAGCTAATACGACTCACTATAGTTACCAAACATAAAGACAGGTTTTAGAGCTAGAAATAGCAAG | Synthesis of *xyl1* sgRNAs |
| TL320_sgRNA_Xyl_3 | AAGCTAATACGACTCACTATAGGCATAAAAGTAATTATCCGGTTTTAGAGCTAGAAATAGCAAG |  |
| TL322_Xyl1_KO_sc_F | ATTTACGTCGGTCACAGCGG | *xyl1* KO check |
| TL323_ Xyl1_KO_sc_R | ACTCTACTCTGCAAACCCGC |  |
| TL345_Xyl_F | CTGCATCTAGGCTGCGCAAT | *xyl1* KO sequencing |
| TL324_ Xyl1_WT_F | CTCGAGTGTTTGGTCCCTCC | *xyl1* WT check |
| TL325_ Xyl1_WT_R | GACGCAACGATATCGGGGAT |  |
| TL_327_Gs1_gRNA6 | AAGCTAATACGACTCACTATAGTCAAGACACCTAGTATCCAAGTTTTAGAGCTAGAAATAGCAAG | Synthesis of *gs1* sgRNAs |
| TL_328_Gs1_gRNA7 | AAGCTAATACGACTCACTATAGAGTGGTAGCTGGTAATCGTGGTTTTAGAGCTAGAAATAGCAAG |  |
| TL_330_Gs1_F | GAGGAAGGGGAGGAAAATCAAGG | *gs1* KO check |
| TL_331_Gs1_R | ACACATGATGTCAGGCTTTGATG |  |
| TL330 | GAGGAAGGGGAGGAAAATCAAGG | *gs1* KO sequencing |
| TL 93 Gs1 Ct FW | ATGCAATATCGGCGCAGATGG | *gs1* WT check |
| TL 94 Gs1 CT RV | GTTGCGACGACATTGACGTAGC |  |
| TL_256_Bot2_gRNA3 | AAGCTAATACGACTCACTATAGCTGCTTCACCTCCTCCGCTGGTTTTAGAGCTAGAAATAGCAAG | Synthesis of *bot2* sgRNAs |
| TL_266_Bot2_gRNA5 | AAGCTAATACGACTCACTATAGACAAAACATCATTCACCCTGGTTTTAGAGCTAGAAATAGCAAG |  |
| TL 91 Bot2 CT FW | GTCATCTCGGCGTCCTGTATG | *bot2* KO check |
| Bot2_RT_rev | CCCTCAGGACCCAAGTAAC |  |
| TL 133 Bot2 KO F | CGACGATCGTACGTTGCTCTTAGTCATTGGACG | *bot2* KO sequencing |
| TL 92 Bot2 CT RV | AAGAACTGGAGCAGTGTTGTC | *bot2* WT check |
| Bot2_B0510_rev | GTGGATAGACCAGCACAAAC |  |
| TL_263_Boa6_gRNA4 | AAGCTAATACGACTCACTATAGACGATAGTAAGCGATACGAAGTTTTAGAGCTAGAAATAGCAAG | Synthesis of *boa6* sgRNAs |
| TL_265_Boa6_gRNA6 | AAGCTAATACGACTCACTATAGAACCAGGTAAACCAGCCATGGTTTTAGAGCTAGAAATAGCAAG |  |
| TL347_Boa6_F | TGCACAATGGCCGGCAATG | *boa6* KO check |
| TL348_Boa6_R | GAGATAGTGGGACGATATGGTCGC |  |
| TL 128 Boa6 KO F | CACTTCGTGAGCTTCCAGATGCTCCGAAATGG | *boa6* KO sequencing |
| TL 90 Boa6 CT RV | CATTGTCTCCACGAGATAGCACTC | *boa6* WT check |
| TL157 Boa6 WT FW | GGGGTCACATTCTCTGCTGTAGTCGG |  |
| TL294_Pg1_gRNA1 | AAGCTAATACGACTCACTATAGTTGATGCGAAATGTTAACAGGTTTTAGAGCTAGAAATAGCAAG | Synthesis of *pg1* sgRNAs |
| TL296_Pg1_gRNA3 | AAGCTAATACGACTCACTATAGAAGAGAAGACTGATAACAGGTTTTAGAGCTAGAAATAGCAAG |  |
| TL298_Pg1_F | GCCCATTCAACAAGAAAGTGGTG | *pg1* KO check |
| TL299_Pg1_R | CCGCTATATTGGCGAATGACAACC |  |
| TL298_Pg1_F | GCCCATTCAACAAGAAAGTGGTG | *pg1* KO sequencing |
| Bcpg1_KO_Confirm_F | ATGGTTCAACTTCTCTCAATG | *pg1* WT check |
| Bcpg1_KO_Confirm_R | GATATCGGAGACAGTGTTGTC |  |
| TL300_Pg2_gRNA1 | AAGCTAATACGACTCACTATAGTGGAGCTGCAGACACCAAAGGTTTTAGAGCTAGAAATAGCAAG | Synthesis of *pg2* sgRNAs |
| TL302_Pg2_gRNA3 | AAGCTAATACGACTCACTATAGACAATGGTTGATAACCATGGTTTTAGAGCTAGAAATAGCAAG |  |
| TL305_Pg2_R | CCACCCAATATCTAACCACCAAATGC | *pg2* KO check |
| TL358_Pg2_L_F | TCCGTATGGTCAGCTCCAGACC |  |
| TL356_Pg2_R_R | ATGACACAGGGTCGTGGGAT |  |
| TL353_Pg2_L_F | TCCATTCCGGCTTCCACTTCG |  |
| TL353 _Pg2_L_F | TCCATTCCGGCTTCCACTTCG | *pg2* KO sequencing |
| Bcpg2_KO_Confirm_FW | ATGGTTCATATCACAAGCCTT | *pg2* WT check |
| Bcpg2_KO_Confirm_RW | TCCACCGGTGAAAGTAATG |  |
