# Supplementary material for: Multiple knockout mutants reveal a high redundancy of phytotoxic compounds contributing to necrotrophic pathogenesis of Botrytis cinerea
Source: PLoS Pathog. 2022 Mar 3;18(3):e1010367. doi: 10.1371/journal.ppat.1010367 (PMC8923502; doi:10.1371/journal.ppat.1010367)
Supplement: S2 Table — (DOCX) [file ppat.1010367.s007.docx]

**S2 Table. Mapping of the deletions in the 12xbb and 12xpg (*pg1* and *pg2* only) mutants.**

|  |  | **Deletion size** | |  |  |  | **12xbb** | **6x** |
| --- | --- | --- | --- | --- | --- | --- | --- | --- |
| **Gene name** | **Protein size** | **Predicted^1^** | **Observed** | **Deletion start** | **Deletion end** | **Codons deleted** | **Indel depth^2^** | |
| Bcin03g00500: *spl1* | 137 aa | 331bp | 330 bp | 3:185839 | 3:186168 | 24-end^3^ | 92 | 76 |
| Bcin06g06720: *nep1* | 246 aa | 398 bp | 398 bp | 6:2351724 | 6:2352121 | 34-end^3^ | 100 | 66 |
| Bcin02g07770: *nep2* | 244 aa | 271 bp | 271 bp | 2:2793732 | 2:2794002 | 80-133 | 110 | 88 |
| Bcin14g01200: *hip1* | 151 aa | 743 bp | 742 bp | 14:538686 | 14:539427 | all | 48 | 30 |
| Bcin03g00480: *xyn11A* | 227 aa | 434 bp | 434 bp | 3:174482 | 3:174915 | 64-end^3^ | 66 | 65 |
| Bcin03g03630: *xyg1* | 248 aa | 1194 bp | 1193 bp | 3:1224165 | 3:1225357 | all | 33 | 31 |
| Bcin10g01020: *plp1* | 147 aa | 565 bp | 564 bp | 10:417793 | 10:418356 | all | 66 | (WT) |
| Bcin15g00100: *ieb1* | 187 aa | 341 bp | 341 bp | 15:78367 | 15:78707 | 20-115 | 98 | (WT) |
| Bcin09g01800: *xyl1* | 329 aa | 1239 bp | 1239 bp | 9:672899 | 9:674137 | all | 32 | (WT) |
| Bcin04g04190: *gs1* | 645 aa | 2344 bp | 2344 bp | 4:1491832 | 4:1494175 | all | 46 | (WT) |
| Bcin12g06390: *bot2* | 399 aa | 588 bp | 589 bp | 12:2224280 | 12:2224868 | 157-end^3^ | 69 | (WT) |
| Bcin01g00060: *boa6* | 2460 aa | 2626 bp | 2626 bp | 1:18020 | 1:20645 | 661-end^3^ | 45 | (WT) |
| Bcin14g00850: *pg1* | 382 aa | 1627 bp | 1625 bp | 14:371071 | 14:372695 | all | (WT) | (WT) |
| Bcin14g00610: pg2 | 374 aa | 1299 bp | 4275 bp*** | 14:245745 | 14:250019 | all | (WT) | (WT) |

^1^ Deletions were predicted based on the expected cleavage sites of each pair of Cas9-RNPs.

^2^ Indel depth determined from genome sequencing, using ITdel software.

^3^ Deletions of codons due to frameshift. ^4^ The 4275 bp deletion extended into Bcin14g00600 encoding a sclerotia-specifically expressed polyketide synthetase. Since this gene is not expressed *in planta*, it is not expected to contribute to infection.
