# Supplementary material for: Multiple knockout mutants reveal a high redundancy of phytotoxic compounds contributing to necrotrophic pathogenesis of Botrytis cinerea
Source: PLoS Pathog. 2022 Mar 3;18(3):e1010367. doi: 10.1371/journal.ppat.1010367 (PMC8923502; doi:10.1371/journal.ppat.1010367)
Supplement: S3 Table — (DOCX) [file ppat.1010367.s008.docx]

**S3 Table. Mutations in 6x and 12xbb mutants revealed by genome sequencing**

| **No.** | **Gene name** | **Position** | **Ref.** | **Alt.** | **Type** | **Aa change (size)** | **Impact** | **12xbb** | | | **6x** | | | **WT** | | |
| --- | --- | --- | --- | --- | --- | --- | --- | --- | --- | --- | --- | --- | --- | --- | --- | --- |
|  |  |  |  |  |  |  |  | **GQ** | **Ref.** | **Alt.** | **GQ** | **Ref.** | **Alt.** | **GQ** | **Ref.** | **Alt.** |
| 1 | Bcin01g03890 | 1:1431774 | A | G | downstream | no | no | 69 | 0 | 25 | 24 | 8 | 0 | 66 | 22 | 0 |
| 2 | Bcin03g03320 | 3:1110870 | A(T)_12_ | A(T)_13_ | upstream | no | no | 56 | 3 | 31 | 66 | 0 | 22 | 58 | 29 | 2 |
| 3 | Bcin03g08690 | 3:2974326 | A | G | missense | Lys272Glu (544) | unknown | 99 | 0 | 87 | 99 | 0 | 57 | 99 | 91 | 0 |
| 4 | Bcin05g01600 | 5:609368 | G | A | upstream | no | no | 99 | 0 | 65 | 99 | 0 | 33 | 99 | 58 | 1 |
| 5 | Bcin06g00550 | 6:240208 | C | T | missense | Ser106Leu (202) | unknown | 99 | 0 | 137 | 99 | 0 | 91 | 99 | 100 | 0 |
| 6 | Bcin08g00960 | 8:392716 | G | A | 5'-UTR | no | no | 99 | 0 | 86 | 99 | 0 | 61 | 99 | 71 | 0 |
| 7 | Bcin13g05880 | 13:2257505 | G | A | downstream | no | no | 15 | 0 | 5 | 27 | 0 | 9 | 21 | 9 | 0 |
| 8 | Bcin12g06810 | 12:2346277 | G | A | stop gained | Gln255* (532) | gene k.o. | 99 | 0 | 79 | 99 | 0 | 67 | 99 | 98 | 0 |
| 9 | Bcin16g02140 | 16:845307 | T | A | missense | Thr120Ser (439) | low | 99 | 0 | 71 | 99 | 0 | 72 | 99 | 64 | 0 |
| 10 | Bcin01g04950 | 1:1785863 | T(A)_10_ | T(A)_9_ | 3'-UTR | no | no | 99 | 0 | 73 | 99 | 0 | 42 | 33 | 49 | 14 |
| 11 | Bcin09g03260 | 9:1162811 | G | A | 5'-UTR | no | no | 99 | 0 | 86 | 99 | 0 | 62 | 99 | 38 | 33 |

Ref.: Reference sequence. Alt: Altered (mutated) sequence. GQ: GQ score for sequencing quality. *Premature translation stop.
