# Supplementary material for: Multiple knockout mutants reveal a high redundancy of phytotoxic compounds contributing to necrotrophic pathogenesis of Botrytis cinerea
Source: PLoS Pathog. 2022 Mar 3;18(3):e1010367. doi: 10.1371/journal.ppat.1010367 (PMC8923502; doi:10.1371/journal.ppat.1010367)
Supplement: S4 Table — (DOCX) [file ppat.1010367.s009.docx]

**S4 Table. MS/MS-detection of CDIPs in the secretomes of *B. cinerea* WT and multiple k.o. mutants**

| **Strain** | **n** |  | **Spl1** | **Nep2** | **Xyn11A** | **Hip1** | **XYG1** | **IEB1** | **Gs1** | **PG1** | **PG2** |
| --- | --- | --- | --- | --- | --- | --- | --- | --- | --- | --- | --- |
| **WT** | 6 | mean | 1,58 | 0,11 | 0,65 | 0,07 | 1,49 | 1,21 | 1,08 | 1,24 | 2,47 |
|  |  | st.dev. | 1,44 | 0,05 | 0,37 | 0,08 | 0,82 | 0,68 | 0,81 | 0,65 | 1,66 |
| **4x^R^** | 5 | mean | 0 | 0 | 0 | 0,03 | 1,32 | 1,06 | 1,13 | 1,31 | 2,75 |
|  |  | st.dev. | 0 | 0 | 0 | 0,07 | 1,04 | 0,68 | 0,42 | 0,72 | 2,03 |
| ***pg1 pg2*** | 4 | mean | 1,28 | 0,10 | 1,03 | 0,07 | 2,78 | 1,69 | 1,85 | 0 | 0 |
|  |  | st.dev. | 1,24 | 0,06 | 0,65 | 0,12 | 1,52 | 0,91 | 1,80 | 0 | 0 |
|  |  |  |  |  |  |  |  |  |  |  |  |
| **WT** | 5 | mean | 2,18 | 0,25 | 2,40 | 0,02 | 1,06 | 1,65 | 1,34 | 2,63 | 1,70 |
|  |  | st.dev. | 0,88 | 0,06 | 0,43 | 0,04 | 0,33 | 0,49 | 0,18 | 0,51 | 0,94 |
| **10x** | 4 | mean | 0 | 0 | 0 | 0 | 0 | 0 | 0 | 3,12 | 0,85 |
|  |  | st.dev. | 0 | 0 | 0 | 0 | 0 | 0 | 0 | 0,82 | 0,04 |
| **11x** | 3 | mean | 0 | 0 | 0 | 0 | 0 | 0 | 0 | 0 | 3,89 |
|  |  | st.dev. | 0 | 0 | 0 | 0 | 0 | 0 | 0 | 0 | 0,87 |
| **12xpg** | 3 | mean | 0 | 0 | 0 | 0 | 0 | 0 | 0 | 0,01 | 0 |
|  |  | st.dev. | 0 | 0 | 0 | 0 | 0 | 0 | 0 | 0,01 | 0 |
| **12xbb** | 3 | mean | 0 | 0 | 0 | 0 | 0 | 0 | 0 | 2,76 | 4,82 |
|  |  | st.dev. | 0 | 0 | 0 | 0 | 0 | 0 | 0 | 0,56 | 0,74 |

Mean LQF intensity values divided by total LQF intensity values of all *B. cinerea* secreted proteins are shown. MS/MS proteome analysis was performed with two batches of samples loaded one after the other, the first batch including WT, 4x^R^ and *pg1 pg2* mutants, and the second batch WT, 10x, 11x, 12xpg and 12xbb mutants. n: Number of independent samples analysed. Values of proteins that have been deleted in the analysed mutants are shown with red background.
