# Supplementary material for: Multiple knockout mutants reveal a high redundancy of phytotoxic compounds contributing to necrotrophic pathogenesis of Botrytis cinerea
Source: PLoS Pathog. 2022 Mar 3;18(3):e1010367. doi: 10.1371/journal.ppat.1010367 (PMC8923502; doi:10.1371/journal.ppat.1010367)
Supplement: Attachment — Submitted filename: Response-to-reviewers_22.12.21.docx [file ppat.1010367.s010.docx]

**Responses to comments of the reviewers (responses in red)**

**Part I - Summary**
Please use this section to discuss strengths/weaknesses of study, novelty/significance, general execution and scholarship.

Reviewer #1: The grey mold fungus releases a large number of relatively non host-specific CDIPs (plant cell death inducing proteins) and toxins during infection. On an overall level, it is an interesting question that how they collectively determine its necrogenic ability. Based on an optimized CRISPR/Cas9 protocol, this study systematically explore the functional complexity of different CDIPs and give us some evidences for host cell death induction during necrotrophic pathogenesis. However, this story did not give us an overall landscape or a clear definition of these 12 CDIPs and phytotoxins during B. cinerea infection, for example, how do they work together during infection or how do they play separate roles during infection? Further more, a key question is that how the authors illustrate the specific roles of PRR-induced cell death for necrotrophic infection? Is the cell death  beneficial or detrimental for B. cinerea infection?

Reviewer #2: Fungal plant pathogens commonly secreted a plethora of highly redundant virulence factors to achieve infection. Elimination of single factors was often found to have either no or only limited effects on their pathogenicity, which leads to the contributions of individual factors difficult to be determined. The Leisen et al manuscript created multiple mutants lacking up to 12 genes that encoding the currently known plant cell death inducing proteins (CDIPs) and phytotoxins in Botrytis cinerea by using an optimized CRISPR/Cas9-based method for marker-free genome editing. Based on these multiple mutants, the manuscript comprehensively evaluated the contributions of these virulence factors to the necrotrophic infection of B. cinerea. I really appreciate this thorough study that could systematically explore the functional redundancy of fungal virulence factors.

Reviewer #3: In this study, to comprehensively evaluate the contributions of plant cell death inducing proteins (CDIPs) and metabolites for B. cinerea infection, the authors optimized a CRISPR/Cas protocol which allowed serial marker-free mutagenesis to generate mutants lacking up to 12 CDIPs. Infection analysis revealed a successive decrease in virulence with increasing numbers of mutated genes, and varying effects of knockouts on different host plants. In addition, the on planta secretomes of the mutants revealed substantial remaining phytotoxic activity, proving that further CDIPs contribute to necrosis and virulence. This study has addressed the functional redundancy of fungal virulence factors, and demonstrates that B. cinerea releases a highly redundant cocktail of proteins to achieve necrotrophic infection of a wide variety of host plants. This work represents the first attempt to systematically explore the functional complexity of fungal virulence factors, which contributes significantly to pathogenesis research on Botrytis and other fungal pathogens.

**Part II – Major Issues: Key Experiments Required for Acceptance**
Please use this section to detail the key new experiments or modifications of existing experiments that should be absolutely required to validate study conclusions.

Generally, there should be no more than 3 such required experiments or major modifications for a "Major Revision" recommendation. If more than 3 experiments are necessary to validate the study conclusions, then you are encouraged to recommend "Reject".

Reviewer #1:

1. Line 313-317. The mutants show stronger reduction on bean leaves and apple fruit. In the discussion, the authors speculate that it may be caused by different sets of matching receptors or targets in different plants species. As shown in Table 1, many PRRs of these CDIPs had been identified, analysis and prediction of these known PRRs can be done in Phaseolus bean, tomato and maize, and apple fruits to explain the observations. Furthermore, plants mutated in BAK1 and SOBIR1 can be included to test whether these functions are associated with PRRs.

R: It is virtually impossible to identify functional homologs of known PRRs in other plant families. PRRs represent large families of paralogous proteins which are more similar within one species than to the best homologs in another, not closely related species.

Indeed, it is of great interest to test the response of plants that are mutated or silenced in PTI signaling components. We now present the results of infections with two tobacco SOBIR1 knock-out lines, and tobacco plants silenced for BAK1 (data in new Fig. 7). Unexpectedly, neither the sobir1 knockout plants nor the plants silenced for bak1 show a significant change in their sensitivity to B. cinerea WT and 12x mutant infection. These results are discussed in the discussion section.

2. Fig 4. All the mutants generated in this study should be included in this experiment. Besides 12xpg and 12xbb, other mutants can give us a clearer comprehension of the functions of different CDIPs during infection. Even though they may not affect the early stages of lesion formation, it will be good control for others. Furthermore, this experiment was conducted in Phaseolus leaves, it is not sufficient to verify that these CDIPs are involved in the early stages of lesion formation before tested in other plants.

R: We have performed microscopic analyses of necrosis induction with all mutants. The results are shown in the new Fig. 4, and commented in the text.

3. Line 321-323 & Fig S3. It seems that multiple knockouts of 12xpg show similar induction with pg1 pg2 double mutant? The authors should compare these two, 12xpg and double mutant and give an explanation for this. And all the infection pictures should be included in Fig S3.

4. Line 323-326 & Fig S4. Does the multiple knockouts of 12xbb show a stronger reduction in virulence than bot2 boa6 double mutant? The authors should compare these two and give an explanation. And all the infection pictures should be included in Fig S4.
R: We have made more experiments to characterize the virulence of the pg1pg2 and the bot2boa6 mutants on all four tissues. We have also compared the infection behaviour of the 12xpg and the pg1pg2, and of the 12xbb and bot2boa6, and have documented this with new pictures in Figs. S3 and S4. To avoid duplication of data from Fig. 3 for 12xpg and 12xbb mutants, quantitative data are shown in Figs. S3/S4 only for pg1pg2 and bot2boa6. Double mutants generally have significantly reduced virulence on all tissues tested. These defects are smaller than the defects of the corresponding 12x mutants, but sometimes not visible if the infection tests have been performed at different times. Furthermore, the pg1pg2 and bot2boa6 mutants were generated with resistance markers, which makes them not perfectly comparable with the marker-free 12x mutants.

5. Line 344-345. “The early stages of lesion formation”, this conclusion is vague. As the authors mentioned in the manuscript, the infection process contains penetration, primary lesion formation, lesion expansion and sporulation. More detailed observations should be provided during infection.

R: (l. 344: ‘These data show that the CDIPs deleted in these mutants are involved in the early stages of lesion formation‘). We have now performed quantitative inoculation tests with killed onion epidermis layers to compare appressorium formation and penetration of the 12x mutants with WT (Fig. 4A, B). We observed increased germ tube lengthts with the mutants, which indicate a slightly delayed penetration. This might be responsible for the delayed necrosis formation observed for 8x, 10x and 12x mutants. Up to now, we can conclude that early stages of lesion formation are delayed to different extents in the mutants. However, the observed differences are only small and would require substantial efforts to be analysed in detail, also because of the variations observed between experiments, as described in the Discussion (l. 718ff.).

6. Line 370-371. The 10x and the 12xbb mutants were generated in this study. Is there any experiment that can verify that these mutants were impaired in synthesizing botrydial and botcinin?

R: We have arranged a collaboration with the group of Isidro Collado (Univ. Cadiz), who has done metabolite studies with the10x and the 12xbb mutants, which confirm the loss of production of both toxins in the 12xbb mutant. The data are presented in the new Fig. 6.

Reviewer #2: The study assayed the infection of mutants by pairwise inoculations of them with wild type on the same leaf or fruit closely together. Since some of the CDIPs may suppress or trigger host immunity, I wonder whether this method of inoculation hinders the infection test results.

R: First, we think that this way of inoculation allows the most precise comparison between WT and mutants. Compared with single inoculations (one or two inoculation sites per leaf with the same strain), pairwise inoculations provide more reproducible results and a more economical regarding the number of plants required. We have never observed that multiple inoculations affect each other, this was confirmed with control experiments on bean leaves and apples with WT and 12x mutants in the last few weeks, which yielded similar results irrespective of the inoculation schemes.

Fig. 2B-D, it is hard to say whether there is a difference for conidiation and formation of sclerotia and infection cushions based on phenotypic photos alone. It is better to show the quantitative data.

R: We have quantified sporulation and sclerotia formation for all mutants, which are now shown in the diagrams of Figs. 2B and 2C.

Reviewer #3: (No Response)

**Part III – Minor Issues: Editorial and Data Presentation Modifications**
Please use this section for editorial suggestions as well as relatively minor modifications of existing data that would enhance clarity.

Reviewer #1:

Line 6. It should be CRISPR/Cas9

R: Corrected throughout the manuscript
Line 12-13. Sentences are difficult to understand. It should be rephrased.
R: The sentence was rephrased as follows (l. 18 ff.): ‘Despite the loss of most of the known phytotoxic compounds, the on planta secretomes of the multiple mutants revealed retained substantial remaining phytotoxic activity, proving that further, as yet unknown CDIPs contribute to necrosis and virulence’
Line 60 “oft act as”, it should be a mistake.
R: Corrected to ‘often act as...’
Line 206 CRISPR/Cas should be “CRISPR/Cas9”the authors should check through all the manuscript for this.
R: Corrected throughout the manuscript.
Line 256. What is abbreviation of “RNP” for?
R: The abbreviation is now explained (ribonucleoprotein complex).
Line 310-312 & Figure 3. To give us a better understanding of effects of multiple knockouts on infection. More mutants should be included in this test. “spl1& and 3x&” shouldn't be excluded. Authors only show the infection pictures of WT, 12xpg and 12xbb, all the infection pictures should presented.
R: We have included the 4x marker-free mutant into Fig. 3. The 3x mutant showed an impaired growth and infection phenotype after being recultivated from the -80°C stock and was therefore excluded; we also left the spl1 mutant out, because a WT-like phenotype of the marker-containing spl1^C^ mutant is shown in Fig. S1.
Line 321 two mutants
R: Corrected (one ‘mutant’ was deleted).
Line 356 “was performed according to” This sentence doesn't seem to end.
R: Corrected.
Line 362-363 No differential protein abundance in the WT and mutant secretomes. More clear explanation should be given for this.
R: For clarification, we have rephrased the sentence as following (l. 511): ‘However, because of the high variability of intensities of the detected secreted proteins between independent samples, compensatory changes were difficult to determine. Analysis of the proteome data using the Perseus bioinformatic platform (33) did not reveal evidences for differential protein abundance in WT and mutant secretomes.’
Figure 5 the title is easy to been mistaken
R: We have rephrased the title of Fig. 5 to (l. 530): ‘Phytotoxic activity of *B. cinerea on planta* secretomes after leaf infiltration’

Figure 5B and 5D. How the error is calculated? It doesn't seem that significant.

R: Indeed the infiltrations showed considerable variation in toxicity. Nevertheless, statistical evaluation (One-way ANOVA and Dunnett´s post hoc test) revealed significant differences between WT and mutant secretomes (Figs. 5B and 5F).

Reviewer #2:

Please indicate how many biological replicates used for MS/MS proteomics.
R: For MS/MS proteomics, between three and six biological replicates (independent secretome samples) were analysed; this has been made clearer now in the legend toTable S3.

How many heterokaryotic transformants were obtained for 11x, 12xpg, and 12xbb? How many heterokaryotic transformants for each genotype were phenotypically characterized? Do they have the same phenotype? This information should be mentioned in the manuscript.
The number of heterokaryotic transformants can be determined in Table 2 by the difference between total and homokaryotic deletions. To make this better comprehensible, we have modified Table 2.

Colonies showing after PCR with primer pairs A1/A2 (Fig. S2) both WT and expected deletion fragments where considered either mixed (edited and non-edited) transformants or heterokaryons, and were therefore excluded from further analyses.

We have performed a phenotypic analysis of 2-5 genetically identical transformants, in order to select the respective transformant to be used for the next round of transformation. This is now described in the Methods (l. 145).

Line 285, “All mutants displayed growth and sporulation similar to WT”. This assertion is not accurate because the 4xR mutant had a statistically significant growth retardation.
R: We agree, and have corrected this statement. (l. 418ff.)
Table 1, What does the “§” mean?

R: For better comprehension, we have modified Table 1 and removed the symbol “§”.

Reviewer #3: I have only some minor comments:
1) In table 2: In the background strain lacking spl1-nep1-nep2, it was difficult (0%) to obtain a double mutant of xyn11A and ieb1. However, in the background straining Δspl1-nep1-nep2-xyn11A-hip1-xyg1, it was quite easy (57%) to generate a double mutant of plp1 and ieb1. Does it mean that some genome regions could be easy or difficult to be edited by the CRISPR/Cas system?
R: Indeed we found differences in the accessibility of the genes for deletion mutagenesis. This could be due to different efficiencies of the gRNAs used (which are difficult to predict by the available search tools), or to genomic regions that are more difficult to be edited, as suggested. In the case of pg2 we confirmed that this gene is difficult to delete, since we obtained only a single mutant (12xpg) containing a deletion in *pg2* (which was larger than expected), and later teste several other gRNAs until we succeed to generate a defined deletion in *pg2*. This aspect is now commented in the text. (l. 366 ff.). In the case of *ieb1*, the variable efficiency of deletion mutagenesis between transformation round 3 and 5 is difficult to explain.
2. Lines 236-238: Since all single deletion mutants showed no significant differences in virulence compared to WT, if it is possible that deletion of one single CDIP leads to overexpression of other CDIPs although the authors did not observed changes at protein translation level determined by the Perseus bioinformatic platform (lines 359-363)?

R: We cannot exclude this possibility. We did not perform transcriptome analyses which would have allowed to test this for all genes encoding secreted proteins. Unfortunately, the variability of the proteome data was too high to allow a clear conclusion of this. This is now commented (l. 511ff.).
